# Myeloid-derived suppressor cells in transplantation: the dawn of cell therapy

**DOI:** 10.1186/s12967-018-1395-9

**Published:** 2018-01-29

**Authors:** Weitao Zhang, Jiawei Li, Guisheng Qi, Guowei Tu, Cheng Yang, Ming Xu

**Affiliations:** 1 Department of Urology, Zhongshan Hospital, Fudan University, Shanghai Key Laboratory of Organ Transplantation, 180 Fenglin Road, Shanghai, 200032 China; 2 Department of Intensive Care Medicine, Zhongshan Hospital, Fudan University, Shanghai, China

**Keywords:** Myeloid-derived suppressor cell (MDSC), Transplantation, Cell therapy, Immunology, Regulation

## Abstract

Myeloid-derived suppressor cells (MDSCs) are a series of innate cells that play a significant role in inhibiting T cell-related responses. This heterogeneous population of immature cells is involved in tumor immunity. Recently, the function and importance of MDSCs in transplantation have garnered the attention of scientists and have become an important focus of transplantation immunology research because MDSCs play a key role in establishing immune tolerance in transplantation. In this review, we summarize recent studies of MDSCs in different types of transplantation. We also focus on the influence of immunosuppressive drugs on MDSCs as well as future obstacles and research directions in this field.

## Introduction of MDSCs

Regulatory myeloid cells are emerging as novel targets for immunosuppressive agents and hold considerable promise as cellular therapeutic agents [1]. Although myeloid-derived suppressor cells (MDSCs) were initially observed in tumor-bearing patients in the 1980s [2, 3], their exact biological role became appreciated in 2000 [4]. MDSCs develop and differentiate from a common myeloid progenitor (CMP). MDSCs play a significant role in tumor growth and progression, metastasis and resistance to different therapies [5]. Under pathological conditions such as cancer [6–8], infection [9, 10] and transplantation [11, 12], the pathway for CMP differentiation into granulocytes, macrophages, and dendritic cells is inhibited, in which case some CMPs may differentiate into MDSCs.

**Fig. 1:**
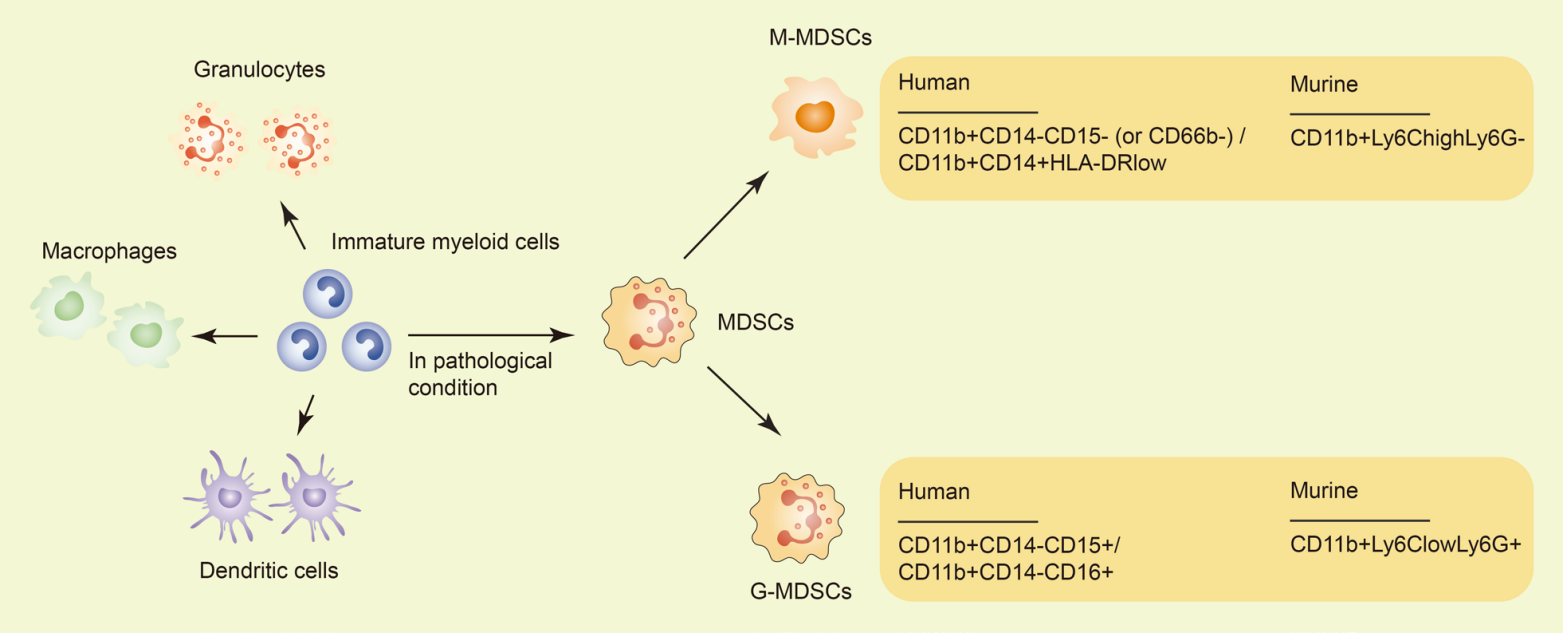
MDSC development and cell subsets in mice and humans. IMCs are part of the normal process of myelopoiesis. IMCs can differentiate into granulocytes, macrophages, and dendritic cells. However, IMCs can also differentiate into MDSCs, especially in some pathological conditions. MDSCs have two major subsets. In mice, G-MDSCs are defined as CD11b^+^Ly6C^low^Ly6G^+^ cells, and M-MDSCs are defined as CD11b^+^Ly6C^high^Ly6G^−^ cells. In humans, G-MDSCs are characterized as CD11b^+^CD14^−^CD15^+^/CD11b^+^CD14^−^CD66^+^ cells, and M-MDSCs are defined as CD11b^+^CD14^−^CD15^−^/CD11b^+^CD14^−^CD66^−^ or CD11b^+^CD14^+^HLA^−^DR^low^ cells

MDSCs are not a terminally differentiated population of cells. In mice, MDSCs are defined as CD11b^+^Gr1^+^ cells. It is now accepted that MDSCs can be divided into two major groups of cells which can be identified by a combination of specific markers. Granulocytic MDSCs (G-MDSCs) are defined as CD11b^+^Ly6C^low^Ly6G^+^ cells and monocytic MDSCs (M-MDSCs) are defined as CD11b^+^Ly6C^high^Ly6G^−^ cells [13–16]. G-MDSCs are the largest population of MDSCs in tumor-bearing mice, representing > 80% of all MDSCs. However, there is no consensus on the definition of MDSC subsets in humans. Historically, human MDSCs were defined as lineage marker (CD3, CD14, CD19, and CD56)-negative, human leukocyte antigen (HLA)-DR-negative and CD33-positive cells purified from mononuclear cells using a Ficoll gradient [17]. More recently, the existence of two subsets of cells (similar to murine models) has been reported in cancer patients, and G-MDSCs are commonly characterized as CD11b^+^CD14^−^ cells expressing a granulocytic marker, CD15 or CD66 [18]. M-MDSCs are defined by two combinations of markers: CD11b^+^CD14^−^CD15^−^ (or CD66b^−^) or CD11b^+^CD14^+^HLA^−^DR^low^ (Fig. 1) [19].

Several types of induction can contribute to the accumulation of MDSCs, including induction by inflammatory cytokines and growth factors (such as granulocyte-macrophage colony stimulating factor (GM-CSF) [20, 21] and interleukin (IL)-6 [22]) and tumor-derived factors (such as vascular endothelial growth factor (VEGF) [23] and transforming growth factor (TGF)-β1 [24]). Furthermore, the functions of MDSCs are highly dependent on the circumstances in which their expansion occurs. Significant cell-mediated immunosuppressive capacities were observed in infectious MDSCs (iMDSCs) and tumor-bearing MDSCs (tMDSCs) in vitro [25].

MDSCs were reported to be endowed with robust immunosuppressive activity in multiple pathophysiological conditions [26, 27]. The crosstalk between MDSCs and immune cells has been illustrated in recent years, including the following aspects: (1) the suppression of T cell proliferation [28] and increased T cell apoptosis [29]; (2) the potential suppression of the B cell reaction via nitric oxide (NO) [30, 31]; (3) the inhibition of dendritic cell development [32, 33]; (4) the impairment of the effect of natural killer cells on alloantigens [34]; (5) promotion expansion of protumorigenic T regulatory cells [35] and (6) the modulation of cytokine production of macrophages [36].

Even though different types of immune cells activity can be suppressed by MDSCs, the primary ‘targets’ of MDSCs are T cells [37]. There are several mechanisms concerning MDSCs’ suppression of T cells: (1) MDSCs suppress T cell proliferation by depletion of l-arginine via high expression of arginase 1 (Arg1) and inducible nitric oxide synthase (iNOS) [38]. (2) MDSCs nitrate T cell receptors and then inhibit their interaction with cognate antigen-MHC complexes by expressing high levels of reactive oxygen species (ROS) [39]. (3) Other factors such as IL-10, B7-H1 and MHC classII are also involved in the suppressive activity of MDSCs [40].

Considering their immunosuppressive function, many studies have reported the significance of MDSCs in transplantation [1, 41, 42], from bench to bedside, in order to establish immune tolerance and to promote the long-term survival of transplants. This review summarizes recent advances on the effect and application of MDSCs on transplantation.

### Solid organ transplantation

#### Kidney transplantation

Kidney transplantation is the most mature and common type of solid organ transplantation worldwide. The first report of MDSCs in an experimental kidney transplantation animal model was in 2008. A rat model of anti-CD28-induced kidney allograft tolerance showed an accumulation of plastic-adherent CD11b^+^ myeloid cells expressing CD80/86 that were defined as MDSCs. This study indicated that MDSCs had nonspecific immunosuppressive activity in vivo and in vitro involving the action of inducible nitric oxide synthase (iNOS), which was upregulated after contact with activated effector T cells but not with regulatory T cells (Tregs) [28]. Dilek et al. analyzed gene expression in blood-derived MDSCs from rat recipients of kidney allografts using DNA microarray [43]. They found that CCL5 (Rantes), a chemokine for Tregs, was strongly downregulated after treatment with a tolerizing regimen. The results indicated the contribution of MDSCs to the establishment of a graft-to-periphery CCL5 gradient in tolerant kidney allograft recipients, which controlled the recruitment of Tregs to the graft where they likely contributed to maintaining tolerance [43].

In the clinic, Hock et al. reported renal transplant recipients had elevated frequencies of circulating CD11b^+^CD33^+^HLA-DR^−^ MDSCs (both M-MDSCs and G-MDSCs) [44]. They further traced the change in MDSCs for 1 year post-transplantation. These observational studies showed that MDSC numbers increased rapidly and peaked following commencement of immunosuppression [45]. Luan et al. also found an increase in CD11b^+^CD33^+^HLA-DR^−^ MDSCs in renal transplant recipients [46]. It is well known that Tregs play a key role in immune tolerance induction and maintenance [47]. The authors found that MDSCs isolated from kidney transplant recipients were highly efficient in suppressing the proliferation of CD4^+^ T cells in mixed leukocyte reactions. In addition, CD11b^+^CD33^+^HLA-DR^−^ MDSCs were capable of expanding Tregs in vitro, and their accumulation over time after transplantation was linearly correlated with an increase in Tregs in vivo. This was the first study to link the presence of MDSCs with the emergence of Tregs in vivo in transplant recipients, and to define the subpopulation of MDSCs derived from transplant recipients responsible for generation of Tregs [46]. The results of Meng et al. also supplement the previous findings of MDSCs in kidney transplantation [48]. In this study, 50 renal transplant recipients with acute T cell-mediated rejection were enrolled. The patients were divided into two groups according to the ratio of MDSCs in the peripheral blood mononuclear cell population tested by flow cytometry. As expected, the 1- and 5-year graft survival rates in the high-MDSC group were 93 and 79%, respectively, but were only 68 and 36%, respectively, in the low-MDSC group. As expected, interferon (IFN)-γ- and tumor necrosis factor (TNF)-α-related renal tissue injury was significantly alleviated in the high-MDSC group compared with the low-MDSC group. Furthermore, IL-17, a strong pro-inflammatory cytokine, was inhibited by Tregs expanded by MDSCs [48]. These results indicated that the number of MDSCs in the renal allograft recipients was associated with long-term graft survival and that MDSCs may regulate the imbalance between Tregs and Th17 cells.

#### Cardiac transplantation

In a prolonged murine cold ischemia time-mediated donor cardiac injury model [49], Gong et al. detected three MDSC subsets, CD11b^+^Gr-1^low^, CD11b^+^Gr-1^int^ and CD11b^+^Gr-1^high^. It should be noted that the definition of MDSC subsets was based on Gr-1, without Ly6C. The findings revealed that CD11b^+^Gr-1^low^ MDSCs had strong suppressive activity. MDSC subsets from the tolerant mice exhibited higher suppressive capacities compared with subsets from naive (untreated) mice. Depletion of Tregs increased peripheral and intragraft CD11b^+^Gr-1^low^ MDSC frequency. Intriguingly, boosting Tregs caused a remarkable increase in CD11b^+^Gr-1^low^ MDSC frequency in the graft, peripheral blood and spleen. This study indicated for the first time the possible interaction between MDSCs and Tregs in cardiac transplantation [49]. Furthermore, Nakamura et al. demonstrated that MDSCs induced by PD-L1 were capable of recruiting Foxp3^+^ Tregs [50].

In addition to focusing on the mechanism of MDSC induction, some studies have also examined the possibility of increasing the induction of MDSCs, which may indicate a possible direction for immune therapy in transplantation. Mammalian target of rapamycin (mTOR) inhibitors are the main immunosuppressive drugs for organ transplant recipients. Nevertheless, the mechanisms by which mTOR inhibitors induce immunosuppression are not fully understood. Interestingly, in a murine cardiac transplantation model, rapamycin treatment led to the recruitment of MDSCs and increased their expression of iNOS [51]. Adoptive transcoronary arterial transfer of MDSCs from rapamycin-treated recipients prolonged allograft survival. Co-administration of rapamycin and the mitogen-activated protein kinase (MEK) inhibitor trametinib reversed rapamycin-mediated MDSC recruitment. Thus, the author concluded that the mTOR and Raf/MEK/extracellular signal regulated kinase (ERK) signaling pathways appeared to play an important role in MDSC expansion [51]. IL-33, which is thought to be able to facilitate Th2 responses, significantly increased CD11b^+^Gr1^int^ M-MDSCs in a chronic cardiac rejection model, reduced antibody-mediated rejection and ultimately prolonged allograft survival [52, 53]. These findings support IL-33-based MDSC therapy in cardiac transplantation. The induction of other molecules, such as IL-6 [54, 55] and IFN-γ [56], has also been tightly associated with MDSCs in cardiac transplant protection.

#### Skin transplantation

Skin transplantation is a convenient animal model for the study of rejection and tolerance. In 2008, a study conducted by Zhang et al. demonstrated the expansion of MDSCs by immunoglobulin-like transcript 2 receptor and its ligands [57]. Moreover, the immunosuppressive function of MDSCs was enhanced by this specific kind of receptor, which led to a better survival rate of alloskin grafts after transplantation. Other studies have illustrated that MDSCs induced by IL-2C or neupogen [58], IL-33 [59], Δ9-tetrahydrocannabinol (via the activation of cannabinoid receptor 1) [60] and TNF-α (via an iNOS-dependent mechanism) [61] were able to suppress T cell proliferation, promote Tregs and induce immune tolerance. Considering the close relationship between MDSCs and Tregs, Adeegbe et al. combined induced-MDSCs, which were shown to be superior to naïve MDSCs, with induced-Tregs to promote transplantation tolerance. The results showed that co-administration of these two regulatory cells had a much better effect on graft survival than administration of either one alone [58], which highlights the combination of MDSCs and Tregs as a potential cell therapy for tolerance induction. In another study, the adoptive transfer of MDSCs prolonged skin graft survival but failed to alter antigen-specific CD8^+^ T cell proliferation and cytotoxicity. The authors attributed this result to the over-activation of donor-specific T cells in the spleen [62]. Furthermore, in order to assess the effect of MDSCs in immunosuppressive treatments, Carretero-Iglesia et al. directly compared the function of three regulatory myeloid cell types in a skin transplant model, including autologous tolerogenic dendritic cells, suppressor macrophages (suppMφs) and MDSCs [63]. They found that these three types of cells perform their roles in T cell inhibition in three different ways. autologous tolerogenic dendritic cells mainly regulate the activation, proliferation and reactivation process of T cells, while suppMφs are thought to be responsible for inducing and expanding Treg cells. However, MDSCs appear to exert their immunosuppressive function through Treg expansion as well as induction of T cell death.

### Bone marrow transplantation (BMT)

The discovery of MDSCs can be attributed to BMT. In 1984, MDSCs were first reported in patients who received BMT [64]. At that time, these cells were called “natural suppressor cells”, and it was indicated in the study that MDSC expansion was actually induced by radiation. Because of the application of various pro-inflammatory cytokines, such as IFN-γ, granulocyte-colony stimulating factor (G-CSF), and IL-1β [65], some animal models have emerged in recent years in which MDSCs accumulate and are activated without radiation [66, 67].

The efficacy of BMT can be limited by graft-versus-host disease (GVHD), and the first use of MDSCs in vitro to suppress major-mismatch driven GVHD was reported by Highfill et al. in 2010. The suppression was attributed to l-arginine depletion by arginase-1 activity. More importantly, Highfill et al. found a new subset of MDSC which was produced by exogenous IL-13 (MDSC-IL-13). MDSC-IL-13 is more potently suppressive and results in arginase-1 up-regulation. Compared to MDSCs, MDSC-IL-13 inhibits GVHD lethality with more efficacy. Indeed, MDSC-IL-13 express high levels of PDL1 which regulates tolerance by PD1-PDL1 interactions [68]. It is known that tumor relapse is a common but severe threat to BMT recipients. In a BMT model, Wang et al. revealed that the additional accumulation of MDSCs (both in the spleen and in peripheral blood) after allogeneic BMT was stimulated by tumor relapse [69]. Thus, MDSCs may also serve as a predictor for tumor relapse after BMT. Consistent with this finding, another clinical investigation observed that MDSC subsets in patients who received a hematopoietic stem cell transplant were positively correlated with T, B, and double-negative T cell numbers after transplantation [70]. This study supports the previous findings in animal models and further analyzed the kinetics of MDSC subsets in clinical work. Koehn et al. summarized recent advances in investigation of MDSC and allogeneic hematopoietic cell transplantation [71].

### Other transplantations

#### Corneal transplantation

Even though the acceptance rates of penetrating keratoplasty are relatively high [72, 73], either inflammation or neovascularization can induce corneal graft rejection and failure [74]. When retroorbitally injected into the recipients immediately after keratoplasty surgery, both iMDSCs and tMDSCs had a suppressive effect on CD4^+^ T cell proliferation, and they improved the histological condition and decreased neovascularization of corneal grafts. However, a supplementary injection of iMDSCs did not cause graft improvements at later stages [75]. This result was partly consistent with a study in which CD11b^+^ cells were induced by lipopolysaccharide (LPS) [76].

#### Islet transplantation

Type 1 diabetes accompanied by diabetic complications usually occurs when patients lose insulin-producing pancreatic β cells. The routine therapy for this disease is the administration of exogenous insulin. However, insulin may not fulfill the function of normal β cells. Pancreas transplantation has been considered to address this situation, but deleterious side effects are unavoidable [77–79]. In recent years, islet transplantation in optimal situations has appeared as an alternative to pancreas transplantation [80, 81]. As a type of cell transplant, islet grafts have promising prospects, and the induction of tolerance to remove the dependency on immunosuppressive drugs is challenging and necessary. Considering the significance of MDSCs in transplantation, Arakawa et al. co-transplanted MDSCs (generated from bone marrow cells cultured with hepatic stellate cells and GM-CSF with islet grafts in diabetic recipient mice [82]. The results showed that the co-transplantation of these two types of cells significantly promoted allograft survival. As expected, MDSCs exerted their inhibitory function on T cells in an iNOS-dependent manner. MDSCs from *inos*-deficient mice failed to protect islet allografts [82], which indicated the key role of iNOS in MDSC-induced tolerance in islet transplantation. The conclusion agreed with a previous study in which MDSCs expressing a high level of arginase-1 enhanced antigen-specific Tregs in the B7H1 pathway [83].

### What is the impact of immunosuppressive agents on MDSCs?

To date, complete tolerance induction has not been achieved in the clinic, so we must consider the influence of immunosuppressive agents on MDSCs. Several studies have recently reported on the influence of immunosuppressants on myeloid cells in transplant models. Cyclosporine (CsA), a typical type of calcineurin inhibitor, is extensively used to prevent anti-allograft rejection in clinical settings [84]. CsA was previously reported to stimulate the accumulation and suppressive function of MDSCs. In a skin transplantation study, CsA treatment upregulated the allograft infiltration of CD11b^+^Gr1^+^ cells [85]. However, the increase in CD11b^+^Gr1^+^ MDSCs was not attributed to their proliferation but rather to their migration induced by CsA. Moreover, it was CD11b^+^Gr1^+^ MDSCs that were critical for CsA prolongation of allograft survival. The authors demonstrated that CsA-induced MDSCs exerted their function in ameliorating the allograft immune response through the calcineurin-NFAT-IDO signaling pathway, and these MDSCs were able to regulate T cell differentiation from Th1 to Th2 and also CD8^+^ T cell differentiation.

Rapamycin, as an mTOR inhibitor, is another immunosuppressive agent. In a cardiac transplant model, rapamycin treatment at a dosage of 3 mg/kg at different times after transplantation promoted the recruitment of MDSCs and enhanced the activity of arginase-1 and iNOS in MDSCs [51]. The results also showed that the MEK inhibitor trametinib could reverse MDSC induction, which indicated that the ERK signaling pathway was important in the expansion of MDSCs. Our group further investigated whether the mTOR signaling pathway regulates MDSC differentiation and immunosuppressive function [86]. We found that inhibiting mTOR signaling by rapamycin regulated the induction of MDSC towards the CD11b^+^Ly6G^+^Ly6C^low^ G-MDSC subset. The ability to suppress T-cell proliferation of both bone marrow-derived CD11b^+^Ly6G^+^Ly6C^low^ G-MDSCs and CD11b^+^Ly6G^−^Ly6C^high^ M-MDSCs was enhanced by mTOR signal inhibition via upregulation of arginase-1 and iNOS expression. However, the impact of rapamycin on MDSCs is still controversial. In a skin transplant model, Wu et al. reported that rapamycin significantly delayed alloskin graft rejection by decreasing the number of M-MDSCs and directly inhibited M-MDSC differentiation in vitro [87]. The reason for these different results might be due to the use of different disease models and will require further investigation.

In addition to the previously referenced studies, glucocorticoid (GC) was reported to expand MDSCs both in vivo and in vitro [88–90]. As a typical synthetic GC immunosuppressant, dexamethasone was chosen to explore the potential relationship between GC and MDSCs. In alloskin transplant recipient mice after dexamethasone treatment, MDSCs prolonged graft survival and acted as functional suppressive immune modulators that resulted in fewer IFN-γ-producing Th1 cells and a greater number of IL-4-producing Th2 cells. Dexamethasone-treated MDSCs promoted reciprocal differentiation between Th1 and Th2 in vivo in a GC receptor-dependent manner [91]. In addition to transplantation, dexamethasone was demonstrated to regulate the suppressive function of MDSCs via hypoxia inducible factor (HIF)-1α as well as by GC receptor-HIF1α glycolysis in an immunological hepatic injury model [91]. High-dosage dexamethasone rescued MDSC numbers and promoted the suppressive function of MDSCs via Ets1 in immune thrombocytopenia patients [92]. Table 1 summarizes recent studies of immunosuppressive agents and MDSCs. However, the effect of other immunosuppressive agents on MDSCs, such as tacrolimus and mycophenolate mofetil, is still unknown.

**Table 1:** The regulation of myeloid-derived suppressor cells (MDSCs) by immunosuppressive drugs

Immunosuppressive drug	Year	Disease model	Induction of MDSCs	Mechanism	Ref.
Cyclosporine A	2014	Skin transplantation	Yes	Calcineurin-NFAT-IDO	[85]
Rapamycin	2015	Cardiac transplantation	Yes	iNOS/Arg-1	[51]
Rapamycin	2017	AKI	Yes	iNOS/Arg-1	[86]
Rapamycin	2015	Skin transplantation	No	iNOS/Arg-1	[87]
Dexamethasone	2014	Skin transplantation	Yes	GC-GR-NO	[91]
Dexamethasone	2017	Immunological hepatic injury	Yes	GC-GR-H1α	[92]

*AKI* acute kidney injury, *IDO* indoleamine 2, 3-dioxygenase, *Arg-1* arginase-1

### Can MDSCs promote or induce immune tolerance?

Currently, no standard tolerance induction protocol is available in routine clinical practice. However, harnessing the tolerogenic potential of immune cell therapy in transplantation, including MDSC-based cell therapy, may provide an opportunity to accomplish this goal. In 2008, Dugast et al. reported an accumulation of MDSCs in the blood of a rat renal transplantation model [28]. These cells were able to inhibit proliferation but not activation of effector T cells and could induce apoptosis in a contact-dependent manner. However, adoptive transfer of MDSCs failed to induce allograft tolerance in recently transplanted recipients. Thus, researchers have focused on how to induce MDSCs that are able to induce immune tolerance. Zhao et al. found that M-CSF- and TNF-α-induced M-MDSCs have powerful immunosuppressive activity in an iNOS-dependent pathway, and that M-CSF + TNF-α-induced M-MDSCs were able to promote immune tolerance to donor antigens in a murine skin transplant model [61]. In addition to transplantation, autoimmune diseases also need antigen-specific immune supersession. In multiple sclerosis, G-MDSCs have been shown to participate in the process of tolerance induction. G-MDSCs were shown to adopt a more suppressive phenotype during peptide immunotherapy and to inhibit CD4^+^ T-cell proliferation in a cell-contact-dependent manner [93].

### Attempts of MDSC-based cell therapy in transplantation

The therapeutic value of MDSCs has been recognized in patients with cancer [94, 95], inflammation [96, 97] and autoimmune disease [62, 98, 99]. In these conditions, MDSCs usually serve as biomarkers, and the program of therapy may focus on blockade of these cells. However, because of the immune suppressive function of this heterogeneous cell population, there has been growing interest in MDSC-based cell therapy in transplantation. Researchers have aimed to manipulate MDSCs to achieve immune tolerance in the context of transplantation. As mentioned previously, the adoptive transfer of MDSCs was first attempted in 2008, although it failed to induce kidney allograft tolerance [28]. Since then, several investigators have confirmed that repeated injection of MDSCs promotes allograft survival in skin [61], corneal [75] and skin-corneal combined transplantations [25]. There have thus far been no reports concerning MDSC infusion in human transplant recipients. In BMT models, transplantation of MDSCs generated from bone marrow cells by GM-CSF/G-CSF in vitro inhibited GVHD-induced death and attenuated histologic GVHD, whereas the antitumor cytotoxicity of alloantigen-specific T cells was maintained [100]. However, the stability of MDSC immune suppressive function requires a certain microenvironment. For example, Koehn et al. reported that transferred MDSCs lost their suppressive function and their potential to inhibit GVHD lethality immediately upon entering a conditioning regimen that subjected them to GVHD inflammatory settings [101]. This indicated that in the BMT setting, the use of MDSCs as a therapeutic approach for preventing GVHD and other systemic inflammatory conditions may be more effective when combined with approaches limiting in vivo MDSC inflammasome activation.

## Conclusion and perspectives

MDSCs were once thought to play a significant role in the mechanism and therapeutic treatment of tumors. Their potential diagnostic value, combined with their therapeutic value in transplantation, has now become the focus of immunologists and clinicians because MDSCs can inhibit immunoreactions. Considering this function, MDSCs may be used to induce immune tolerance and prolong allograft survival in clinical applications of the future. However, until recently, the differentiation of MDSCs has not been efficient, which has made their application difficult [102]. In addition, it is still unknown whether G-MDSCs and M-MDSCs are terminally differentiated subsets of MDSCs, or whether the phenotype and function of MDSCs subsets are stable. Macrophages, which are also differentiated from immature myeloid cells like MDSCs, consist of M1 and M2 subsets which can convert into each other in some immune microenvironments [103, 104]. We therefore cannot exclude the possibility that G-MDSCs and M-MDSCs can similarly convert into each other. As Scalea et al. pointed, it remains unknown if the type of organ transplanted (e.g. kidney versus liver) leads to MDSCs with different suppressive capacities [42].

**Fig. 2:**
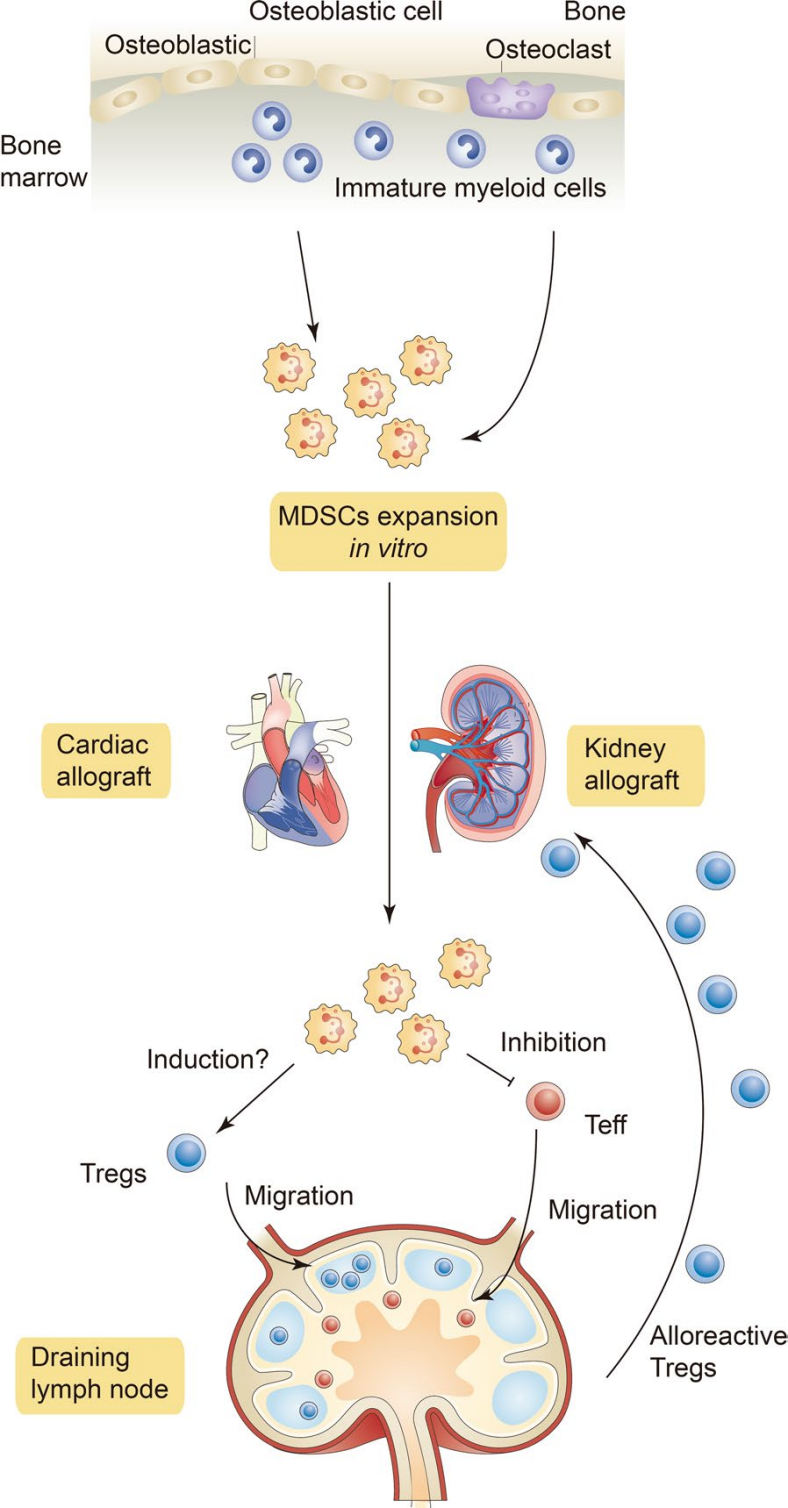
MDSC cell therapy. MDSCs from the bone marrow of recipients might be expanded in vitro and infused into allograft recipients. As immune suppressive innate immunocytes, MDSCs inhibit effector T cell (Teff) function. MDSCs might induce Tregs). The Teffs and Tregs migrate into draining lymph nodes and generate alloreactive Tregs. Alloreactive Tregs are further recruited to allografts where they inhibit rejection and induce tolerance

There are many ideas on how to manipulate the differentiation and purification of MDSCs to make them more useful. In addition, the particular markers for MDSCs, the inductive pathways of MDSCs and the molecular mechanisms regulating MDSCs still need to be identified to evaluate the specific properties of MDSCs. Recent studies have been restricted to animal models or in vitro studies of the molecular mechanisms, but future investigations are expected to be conducted in humans within a safe environment. Like Tregs [105], manipulation of MDSCs in vitro and infusion MDSCs into allograft recipients as a form of cell therapy will require more animal studies before going to clinical trial (Fig. 2). The specific surface markers, stability, lifespan and molecular effector pathways/mechanisms of MDSCs need to be fully identified. Moreover, the efficiency, specificity and safety of MDSC-mediated treatment remain to be determined with experimental and preclinical studies. We believe that by solving the above difficulties, the application of MDSCs in transplantation will be significantly advanced in the future.

## Abbreviations

MDSCs: myeloid-derived suppressor cells
CMP: common myeloid progenitor
GM-CSF: granulocyte-macrophage colony stimulating factor
TGF: transforming growth factor
Tregs: regulatory T cells
IFN: interferon
TNF: tumor necrosis factor
mTOR: mammalian target of rapamycin
MEK: mitogen-activated protein kinase
ERK: kinaseRaf/MEK/extracellular signal regulated kinase
suppMφs: suppressor macrophages
BMT: bone marrow transplantation
GVHD: graft-versus-host disease
Arg1: arginase-1
LPS: lipopolysaccharide
CsA: cyclosporine
HIF: hypoxia inducible factor

## References

[1] Rosborough BR, Raich-Regue D, Turnquist HR, Thomson AW (2014). Regulatory myeloid cells in transplantation. Transplantation.

[2] Buessow SC, Paul RD, Lopez DM (1984). Influence of mammary tumor progression on phenotype and function of spleen and in situ lymphocytes in mice 2. J Natl Cancer Inst.

[3] Young MR, Newby M, Wepsic HT (1987). Hematopoiesis and suppressor bone marrow cells in mice bearing large metastatic Lewis lung carcinoma tumors. Cancer Res.

[4] Bronte V, Apolloni E, Cabrelle A, Ronca R, Serafini P, Zamboni P, Restifo NP, Zanovello P (2000). Identification of a CD11b(+)/Gr-1(+)/CD31(+) myeloid progenitor capable of activating or suppressing CD8(+) T cells. Blood.

[5] Ibanez-Vea M, Zuazo M, Gato M, Arasanz H, Fernandez-Hinojal G, Escors D, Kochan G (2017). Myeloid-derived suppressor cells in the tumor microenvironment: current knowledge and future perspectives. Arch Immunol Ther Exp.

[6] Haskill S, Koren H, Becker S, Fowler W, Walton L (1982). Mononuclear-cell infiltration in ovarian cancer. III. Suppressor-cell and ADCC activity of macrophages from ascitic and solid ovarian tumours. Br J Cancer.

[7] Martinez-Bosch N, Vinaixa J, Navarro P. Immune evasion in pancreatic cancer: from mechanisms to therapy. Cancers. 2018;10(1). 10.3390/cancers10010006. 10.3390/cancers10010006 PMC5789356 29301364

[8] Hanna BS, Ozturk S, Seiffert M (2017). Beyond bystanders: myeloid cells in chronic lymphocytic leukemia. Mol Immunol.

[9] Bunt SK, Clements VK, Hanson EM, Sinha P, Ostrand-Rosenberg S (2009). Inflammation enhances myeloid-derived suppressor cell cross-talk by signaling through Toll-like receptor 4. J Leukoc Biol.

[10] Mishra PK, Morris EG, Garcia JA, Cardona AE, Teale JM (2013). Increased accumulation of regulatory granulocytic myeloid cells in mannose receptor C type 1-deficient mice correlates with protection in a mouse model of neurocysticercosis. Infect Immun.

[11] Wu T, Zhao Y, Zhao Y (2014). The roles of myeloid-derived suppressor cells in transplantation. Expert Rev Clin Immunol.

[12] Ochando JC, Chen SH (2012). Myeloid-derived suppressor cells in transplantation and cancer. Immunol Res.

[13] Zhang C, Wang S, Liu Y, Yang C (2016). Epigenetics in myeloid derived suppressor cells: a sheathed sword towards cancer. Oncotarget.

[14] Fleming TJ, Fleming ML, Malek TR (1993). Selective expression of Ly-6G on myeloid lineage cells in mouse bone marrow. RB6-8C5 mAb to granulocyte-differentiation antigen (Gr-1) detects members of the Ly-6 family. J Immunol.

[15] Youn JI, Nagaraj S, Collazo M, Gabrilovich DI (2008). Subsets of myeloid-derived suppressor cells in tumor-bearing mice. J Immunol.

[16] Bronte V, Brandau S, Chen S, Colombo M, Frey A, Greten T, Mandruzzato S, Murray P, Ochoa A, Ostrand-Rosenberg S (2016). Recommendations for myeloid-derived suppressor cell nomenclature and characterization standards. Nat Commun.

[17] Talmadge JE, Gabrilovich DI (2013). History of myeloid-derived suppressor cells. Nat Rev Cancer.

[18] Brandau S, Trellakis S, Bruderek K, Schmaltz D, Steller G, Elian M, Suttmann H, Schenck M, Welling J, Zabel P (2011). Myeloid-derived suppressor cells in the peripheral blood of cancer patients contain a subset of immature neutrophils with impaired migratory properties. J Leukoc Biol.

[19] Rodriguez PC, Ernstoff MS, Hernandez C, Atkins M, Zabaleta J, Sierra R, Ochoa AC (2009). Arginase I-producing myeloid-derived suppressor cells in renal cell carcinoma are a subpopulation of activated granulocytes. Can Res.

[20] Rößner S, Voigtländer C, Wiethe C, Hänig J, Seifarth C, Lutz MB (2005). Myeloid dendritic cell precursors generated from bone marrow suppress T cell responses via cell contact and nitric oxide production in vitro. Eur J Immunol.

[21] Morales J, Kmieciak M, Knutson K, Bear H, Manjili M (2010). GM-CSF is one of the main breast tumor-derived soluble factors involved in the differentiation of CD11b-Gr1-bone marrow progenitor cells into myeloid-derived suppressor cells. Breast Cancer Res Treat.

[22] Bunt SK, Yang L, Sinha P, Clements VK, Leips J, Ostrand-Rosenberg S (2007). Reduced inflammation in the tumor microenvironment delays the accumulation of myeloid-derived suppressor cells and limits tumor progression. Can Res.

[23] Melani C, Chiodoni C, Forni G, Colombo MP (2003). Myeloid cell expansion elicited by the progression of spontaneous mammary carcinomas in c-erbB-2 transgenic BALB/c mice suppresses immune reactivity. Blood.

[24] Yang L, Huang J, Ren X, Gorska AE, Chytil A, Aakre M, Carbone DP, Matrisian LM, Richmond A, Lin PC (2008). Abrogation of TGFβ signaling in mammary carcinomas recruits Gr-1^+^ CD11b^+^ myeloid cells that promote metastasis. Cancer Cell.

[25] He Y, Bei J, Zeng H, Pan Z (2016). The roles of sepsis-induced myeloid derived suppressor cells in mice corneal, skin and combined transplantation. Transpl Immunol.

[26] Zhang C, Wang S, Yang C, Rong R. The crosstalk between myeloid derived suppressor cells and immune cells: to establish immune tolerance in transplantation. J Immunol Res 2016;2016: 4986797. Published online 2016 Oct 27. 10.1155/2016/4986797. 10.1155/2016/4986797 PMC5102737 27868073

[27] Tamadaho R, Hoerauf A, Layland L. Immunomodulatory effects of myeloid-derived suppressor cells in diseases: role in cancer and infections. Immunobiology. 2017. 10.1016/j.imbio.2017.07.001. 10.1016/j.imbio.2017.07.001 29246400

[28] Dugast A-S, Haudebourg T, Coulon F, Heslan M, Haspot F, Poirier N, de Silly RV, Usal C, Smit H, Martinet B (2008). Myeloid-derived suppressor cells accumulate in kidney allograft tolerance and specifically suppress effector T cell expansion. J Immunol.

[29] Bronte V, Wang M, Overwijk WW, Surman DR, Pericle F, Rosenberg SA, Restifo NP (1998). Apoptotic death of CD8^+^ T lymphocytes after immunization: induction of a suppressive population of Mac-1^+^/Gr-1^+^ cells. J Immunol.

[30] Green KA, Cook WJ, Green WR (2013). Myeloid-derived suppressor cells in murine retrovirus-induced AIDS inhibit T-and B-cell responses in vitro that are used to define the immunodeficiency. J Virol.

[31] Lelis F, Jaufmann J, Singh A, Fromm K, Teschner A, Pöschel S, Schäfer I, Beer-Hammer S, Rieber N, Hartl D (2017). Myeloid-derived suppressor cells modulate B-cell responses. Immunol Lett.

[32] Rolinski J, Hus I (2014). Breaking immunotolerance of tumors: a new perspective for dendritic cell therapy. J Immunotoxicol.

[33] Poschke I, Mao Y, Adamson L, Salazar-Onfray F, Masucci G, Kiessling R (2012). Myeloid-derived suppressor cells impair the quality of dendritic cell vaccines. Cancer Immunol Immunother.

[34] Li H, Han Y, Guo Q, Zhang M, Cao X (2009). Cancer-expanded myeloid-derived suppressor cells induce anergy of NK cells through membrane-bound TGF-β1. J Immunol.

[35] Chesney JA, Mitchell RA, Yaddanapudi K (2017). Myeloid-derived suppressor cells-a new therapeutic target to overcome resistance to cancer immunotherapy. J Leukoc Biol.

[36] Sinha P, Clements VK, Bunt SK, Albelda SM, Ostrand-Rosenberg S (2007). Cross-talk between myeloid-derived suppressor cells and macrophages subverts tumor immunity toward a type 2 response. J Immunol.

[37] Gabrilovich D (2017). Myeloid-derived suppressor cells. Cancer Immunol Res.

[38] Gabrilovich D, Nagaraj S (2009). Myeloid-derived suppressor cells as regulators of the immune system. Nat Rev Immunol.

[39] Nagaraj S, Gupta K, Pisarev V, Kinarsky L, Sherman S, Kang L, Herber D, Schneck J, Gabrilovich D (2007). Altered recognition of antigen is a mechanism of CD8^+^ T cell tolerance in cancer. Nat Med.

[40] Pinton L, Solito S, Damuzzo V, Francescato S, Pozzuoli A, Berizzi A, Mocellin S, Rossi C, Bronte V, Mandruzzato S (2016). Activated T cells sustain myeloid-derived suppressor cell-mediated immune suppression. Oncotarget.

[41] Ochando J, Conde P, Bronte V (2015). Monocyte-derived suppressor cells in transplantation. Curr Transplant Rep.

[42] Scalea JR, Lee Y, Davila E, Bromberg JS (2017). Myeloid-derived suppressor cells and their potential application in transplantation. Transplantation..

[43] Dilek N, Poirier N, Usal C, Martinet B, Blancho G, Vanhove B (2012). Control of transplant tolerance and intragraft regulatory T cell localization by myeloid-derived suppressor cells and CCL5. J Immunol.

[44] Hock BD, Mackenzie KA, Cross NB, Taylor KG, Currie MJ, Robinson BA, Simcock JW, McKenzie JL (2012). Renal transplant recipients have elevated frequencies of circulating myeloid-derived suppressor cells. Nephrol Dial Transplant.

[45] Hock BD, McKenzie JL, Cross NB, Currie MJ (2015). Dynamic changes in myeloid derived suppressor cell subsets following renal transplant: a prospective study. Transpl Immunol.

[46] Luan Y, Mosheir E, Menon M, Wilson D, Woytovich C, Ochando J, Murphy B (2013). Monocytic myeloid-derived suppressor cells accumulate in renal transplant patients and mediate CD4^+^ Foxp3^+^ Treg expansion. Am J Transplant.

[47] Walsh P, Taylor D, Turka L (2004). Tregs and transplantation tolerance. J Clin Investig.

[48] Meng F, Chen S, Guo X, Chen Z, Huang X, Lai Y, Lin M (2014). Clinical significance of myeloid-derived suppressor cells in human renal transplantation with acute T cell-mediated rejection. Inflammation.

[49] Gong W, Ge F, Liu D, Wu Y, Liu F, Kim BS, Huang T, Koulmanda M, Robson SC, Strom TB (2014). Role of myeloid-derived suppressor cells in mouse pre-sensitized cardiac transplant model. Clin Immunol.

[50] Nakamura T, Nakao T, Ashihara E, Yoshimura N. Myeloid-derived suppressor cells recruit CD4^+^/Foxp3^+^ regulatory T cells in a murine cardiac allograft. Transplant Proc. 2016;48(4):1275–8. 10.1016/j.transproceed.2015.10.060 27320602

[51] Nakamura T, Nakao T, Yoshimura N, Ashihara E (2015). Rapamycin prolongs cardiac allograft survival in a mouse model by inducing myeloid-derived suppressor cells. Am J Transplant.

[52] Turnquist HR, Zhao Z, Rosborough BR, Liu Q, Castellaneta A, Isse K, Wang Z, Lang M, Stolz DB, Zheng XX (2011). IL-33 expands suppressive CD11b^+^ Gr-1^int^ and regulatory T cells, including ST2L^+^ Foxp3^+^ cells, and mediates regulatory T cell-dependent promotion of cardiac allograft survival. J Immunol.

[53] Brunner SM, Schiechl G, Falk W, Schlitt HJ, Geissler EK, Fichtner-Feigl S (2011). Interleukin-33 prolongs allograft survival during chronic cardiac rejection. Transpl Int.

[54] Ge F, Yuan S, Su L, Shen Z, He A, Huang T, Gong W (2013). Alteration of innate immunity by donor IL-6 deficiency in a presensitized heart transplant model. PLoS ONE.

[55] Gong W, Shou D, Cheng F, Shi J, Ge F, Liu D (2015). Tolerance induced by IL-6 deficient donor heart is significantly involved in myeloid-derived suppressor cells (MDSCs). Transpl Immunol.

[56] Bryant J, Lerret NM, Wang J-J, Kang H-K, Tasch J, Zhang Z, Luo X (2014). Preemptive donor apoptotic cell infusions induce IFN-γ-producing myeloid-derived suppressor cells for cardiac allograft protection. J Immunol.

[57] Zhang W, Liang S, Wu J, Horuzsko A (2008). Human inhibitory receptor ILT2 amplifies CD11b^+^ Gr1^+^ myeloid-derived suppressor cells that promote long-term survival of allografts. Transplantation.

[58] Adeegbe D, Serafini P, Bronte V, Zoso A, Ricordi C, Inverardi L (2011). In vivo induction of myeloid suppressor cells and CD4^+^ Foxp3^+^ T regulatory cells prolongs skin allograft survival in mice. Cell Transplant.

[59] Gajardo T, Morales RA, Campos-Mora M, Campos-Acuña J, Pino-Lagos K (2015). Exogenous interleukin-33 targets myeloid-derived suppressor cells and generates periphery-induced Foxp3^+^ regulatory T cells in skin-transplanted mice. Immunology.

[60] Sido JM, Nagarkatti PS, Nagarkatti M (2015). Δ9-Tetrahydrocannabinol attenuates allogeneic host-versus-graft response and delays skin graft rejection through activation of cannabinoid receptor 1 and induction of myeloid-derived suppressor cells. J Leukoc Biol.

[61] Yang F, Li Y, Wu T, Na N, Zhao Y, Li W, Han C, Zhang L, Lu J, Zhao Y (2016). TNFα-induced M-MDSCs promote transplant immune tolerance via nitric oxide. J Mol Med.

[62] Drujont L, Carretero-Iglesia L, Bouchet-Delbos L, Beriou G, Merieau E, Hill M, Delneste Y, Cuturi MC, Louvet C (2014). Evaluation of the therapeutic potential of bone marrow-derived myeloid suppressor cell (MDSC) adoptive transfer in mouse models of autoimmunity and allograft rejection. PLoS ONE.

[63] Carretero-Iglesia L, Bouchet-Delbos L, Louvet C, Drujont L, Segovia M, Merieau E, Chiffoleau E, Josien R, Hill M, Cuturi M-C (2016). Comparative study of the immunoregulatory capacity of in vitro generated tolerogenic dendritic cells, suppressor macrophages, and myeloid-derived suppressor cells. Transplantation.

[64] Strober S (1984). Natural suppressor (NS) cells, neonatal tolerance, and total lymphoid irradiation: exploring obscure relationships. Annu Rev Immunol.

[65] Singh VK, Fatanmi OO, Singh PK, Whitnall MH (2012). Role of radiation-induced granulocyte colony-stimulating factor in recovery from whole body gamma-irradiation. Cytokine.

[66] Luyckx A, Schouppe E, Rutgeerts O, Lenaerts C, Koks C, Fevery S, Devos T, Dierickx D, Waer M, Van Ginderachter J (2012). Subset characterization of myeloid-derived suppressor cells arising during induction of BM chimerism in mice. Bone Marrow Transplant.

[67] Sprangers B, Van Wijmeersch B, Luyckx A, Sagaert X, Verbinnen B, Rutgeerts O, Lenaerts C, Tousseyn T, Dubois B, Waer M (2011). Subclinical GvHD in non-irradiated F1 hybrids: severe lymphoid-tissue GvHD causing prolonged immune dysfunction. Bone Marrow Transplant.

[68] Highfill SL, Rodriguez PC, Zhou Q, Goetz CA, Koehn BH, Veenstra R, Taylor PA, Panoskaltsis-Mortari A, Serody JS, Munn DH (2010). Bone marrow myeloid-derived suppressor cells (MDSCs) inhibit graft-versus-host disease (GVHD) via an arginase-1-dependent mechanism that is up-regulated by interleukin-13. Blood.

[69] Wang D, Yu Y, Haarberg K, Fu J, Kaosaard K, Nagaraj S, Anasetti C, Gabrilovich D, Yu X-Z (2013). Dynamic change and impact of myeloid-derived suppressor cells in allogeneic bone marrow transplantation in mice. Biol Blood Marrow Transplant.

[70] Guan Q, Blankstein AR, Anjos K, Synova O, Tulloch M, Giftakis A, Yang B, Lambert P, Peng Z, Cuvelier GD (2015). Functional myeloid-derived suppressor cell subsets recover rapidly after allogeneic hematopoietic stem/progenitor cell transplantation. Biol Blood Marrow Transplant.

[71] Koehn BH, Blazar BR (2017). Role of myeloid-derived suppressor cells in allogeneic hematopoietic cell transplantation. J Leukoc Biol.

[72] Lass JH, Benetz BA, Gal RL, Kollman C, Raghinaru D, Dontchev M, Mannis MJ, Holland EJ, Chow C, McCoy K (2013). Donor age and factors related to endothelial cell loss 10 years after penetrating keratoplasty: specular Microscopy Ancillary Study. Ophthalmology.

[73] Ing JJ, Ing HH, Nelson LR, Hodge DO, Bourne WM (1998). Ten-year postoperative results of penetrating keratoplasty. Ophthalmology.

[74] Bachmann B, Taylor RS, Cursiefen C (2010). Corneal neovascularization as a risk factor for graft failure and rejection after keratoplasty: an evidence-based meta-analysis. Ophthalmology.

[75] He Y, Wang B, Jia B, Guan J, Zeng H, Pan Z (2015). Effects of adoptive transferring different sources of myeloid-derived suppressor cells in mice corneal transplant survival. Transplantation.

[76] Han Y, Zhao S (2015). Protection by LPS-induced inhibitory CD11b^+^ cells on corneal allograft. Int J Clin Exp Med.

[77] Gruessner AC, Sutherland DE (2005). Pancreas transplant outcomes for United States (US) and non-US cases as reported to the United Network for Organ Sharing (UNOS) and the International Pancreas Transplant Registry (IPTR) as of June 2004. Clin Transplant.

[78] White SA, Shaw JA, Sutherland DE (2009). Pancreas transplantation. Lancet.

[79] Troppmann C (2010). Complications after pancreas transplantation. Curr Opin Organ Transplant.

[80] Shapiro AJ (2011). State of the art of clinical islet transplantation and novel protocols of immunosuppression. Curr Diab Rep.

[81] Gibly R, Graham J, Luo X, Lowe W, Hering B, Shea L (2011). Advancing islet transplantation: from engraftment to the immune response. Diabetologia.

[82] Arakawa Y, Qin J, Chou H-S, Bhatt S, Wang L, Stuehr D, Ghosh A, Fung JJ, Lu L, Qian S (2014). Co-transplantation with myeloid-derived suppressor cells protects cell transplants: a crucial role of inducible nitric oxide synthase. Transplantation.

[83] Chou H-S, Hsieh C-C, Charles R, Wang L, Wagner T, Fung JJ, Qian S, Lu L (2012). Myeloid-derived suppressor cells (MDSC) protect islet transplants via B7-H1 mediated enhancement of T regulatory cells. Transplantation.

[84] Kelly P, Kahan BD (2002). Review: metabolism of immunosuppressant drugs. Curr Drug Metab.

[85] Wang X, Bi Y, Xue L, Liao J, Chen X, Lu Y, Zhang Z, Wang J, Liu H, Yang H (2015). The calcineurin-NFAT axis controls allograft immunity in myeloid-derived suppressor cells through reprogramming T cell differentiation. Mol Cell Biol.

[86] Zhang C, Wang S, Li J, Zhang W, Zheng L, Yang C, Zhu T, Rong R (2017). The mTOR signal regulates myeloid-derived suppressor cells differentiation and immunosuppressive function in acute kidney injury. Cell Death Dis.

[87] Wu T, Zhao Y, Wang H, Shao L, Wang R, Lu J, Yang Z, Wang J, Zhao Y (2016). mTOR masters monocytic myeloid-derived suppressor cells in mice with allografts or tumors. Sci Rep.

[88] Varga G, Ehrchen J, Tsianakas A, Tenbrock K, Rattenholl A, Seeliger S, Mack M, Roth J, Sunderkoetter C (2008). Glucocorticoids induce an activated, anti-inflammatory monocyte subset in mice that resembles myeloid-derived suppressor cells. J Leukoc Biol.

[89] Ehrchen J, Steinmüller L, Barczyk K, Tenbrock K, Nacken W, Eisenacher M, Nordhues U, Sorg C, Sunderkötter C, Roth J (2007). Glucocorticoids induce differentiation of a specifically activated, anti-inflammatory subtype of human monocytes. Blood.

[90] Zhang K, Bai X, Li R, Xiao Z, Chen J, Yang F, Li Z (2012). Endogenous glucocorticoids promote the expansion of myeloid-derived suppressor cells in a murine model of trauma. Int J Mol Med.

[91] Liao J, Wang X, Bi Y, Shen B, Shao K, Yang H, Lu Y, Zhang Z, Chen X, Liu H (2014). Dexamethasone potentiates myeloid-derived suppressor cell function in prolonging allograft survival through nitric oxide. J Leukoc Biol.

[92] Lu Y, Liu H, Bi Y, Yang H, Li Y, Wang J, Zhang Z, Wang Y, Li C, Jia A (2017). Glucocorticoid receptor promotes the function of myeloid-derived suppressor cells by suppressing HIF1α-dependent glycolysis. Cell Mol Immunol.

[93] Wegner A, Verhagen J, Wraith DC (2017). Myeloid-derived suppressor cells mediate tolerance induction in autoimmune disease. Immunology.

[94] Baniyash M (2016). Myeloid-derived suppressor cells as intruders and targets: clinical implications in cancer therapy. Cancer Immunol Immunother.

[95] Holmgaard RB, Zamarin D, Li Y, Gasmi B, Munn DH, Allison JP, Merghoub T, Wolchok JD (2015). Tumor-expressed IDO recruits and activates MDSCs in a Treg-dependent manner. Cell Rep.

[96] Guan Q, Moreno S, Qing G, Weiss CR, Lu L, Bernstein CN, Warrington RJ, Ma Y, Peng Z (2013). The role and potential therapeutic application of myeloid-derived suppressor cells in TNBS-induced colitis. J Leukoc Biol.

[97] Zhang H, Lian M, Zhang J, Bian Z, Tang R, Miao Q, Peng Y, Fang J, You Z, Invernizzi P (2018). The functional characteristics CCNI modulation of myeloid-derived suppressor cells in liver inflammation. Hepatology.

[98] Kurkó J, Vida A, Ocskó T, Tryniszewska B, Rauch TA, Glant TT, Szekanecz Z, Mikecz K (2014). Suppression of proteoglycan-induced autoimmune arthritis by myeloid-derived suppressor cells generated in vitro from murine bone marrow. PLoS ONE.

[99] Casacuberta-Serra S, Costa C, Eixarch H, Mansilla M, López-Estévez S, Martorell L, Parés M, Montalban X, Espejo C, Barquinero J (2016). Myeloid-derived suppressor cells expressing a self-antigen ameliorate experimental autoimmune encephalomyelitis. Exp Neurol.

[100] Messmann JJ, Reisser T, Leithauser F, Lutz MB, Debatin KM, Strauss G (2015). In vitro-generated MDSCs prevent murine GVHD by inducing type 2 T cells without disabling antitumor cytotoxicity. Blood.

[101] Koehn BH, Apostolova P, Haverkamp JM, Miller JS, McCullar V, Tolar J, Munn DH, Murphy WJ, Brickey WJ, Serody JS (2015). GVHD-associated, inflammasome-mediated loss of function in adoptively transferred myeloid-derived suppressor cells. Blood.

[102] Escors D, Liechtenstein T, Perez-Janices N, Schwarze J, Dufait I, Goyvaerts C, Lanna A, Arce F, Blanco-Luquin I, Kochan G (2013). Assessing T-cell responses in anticancer immunotherapy: dendritic cells or myeloid-derived suppressor cells?. Oncoimmunology.

[103] Zhou Y, Yu X, Chen H, Sjöberg S, Roux J, Zhang L, Ivoulsou A, Bensaid F, Liu C, Liu J (2015). Leptin deficiency shifts mast cells toward anti-inflammatory actions and protects mice from obesity and diabetes by polarizing M2 macrophages. Cell Metab.

[104] Adamson S, Griffiths R, Moravec R, Senthivinayagam S, Montgomery G, Chen W, Han J, Sharma P, Mullins G, Gorski S (2016). Disabled homolog 2 controls macrophage phenotypic polarization and adipose tissue inflammation. J Clin Investig.

[105] Issa F, Wood KJ (2014). The potential role for regulatory T-cell therapy in vascularized composite allograft transplantation. Curr Opin Organ Transplant.

